# The optimization system for preparation of TG1 competent cells and electrotransformation

**DOI:** 10.1002/mbo3.1043

**Published:** 2020-05-11

**Authors:** Dafei Chai, Gang Wang, Lin Fang, Huizhong Li, Shanshan Liu, Haiying Zhu, Junnian Zheng

**Affiliations:** ^1^ Cancer Institute Xuzhou Medical University Xuzhou Jiangsu P.R. China; ^2^ Center of Clinical Oncology, Affiliated Hospital of Xuzhou Medical University Xuzhou Medical University Xuzhou Jiangsu P.R. China

**Keywords:** electrocompetent cells, electrotransformation, phage display library, TG1, transformation efficiency

## Abstract

An efficient electrotransformation system that includes electrocompetent cells is a critical component for the success of large‐scale gene transduction and replication. The conditions of TG1 competent cell preparation and optimal electrotransformation were evaluated by investigating different parameters. Certain parameters for preparation of TG1 competent cells (≥8 × 10^10^ colony forming units (cfu)/μg DNA) include optimum culture time of monoclonal bacteria (8–10 hr), amplification growth concentration (approximately OD_600_ = 0.45), and culture volume (400 ml in 2 L conical flask). With increased storage of competent cells at −80°C, electrotransformation efficiency gradually decreased, but it remains greater than ≥ 10^10^ cfu/μg DNA 3 months later. Moreover, the recovery time of electrotransformation also influenced electrotransformation efficiency (1.5–2 hr for optimization). The optimized transformation efficiency of TG1 (≥8 × 10^10^ cfu/μg DNA) was observed under suitable electric voltage (2.5 kV), electric intensity (15 kV/cm), and electric time (3.5 ms) of electricity for plasmid transformation. Optimized DNA amount (0.01–100 ng) dissolved in water led to the high efficiency of plasmid transformation (≥8 × 10^10^ cfu/μg DNA), but had low efficiency when dissolved in T4 ligation buffer (≤3 × 10^10^ cfu/μg DNA). These results indicated that an optimized TG1 transformation system is useful for high electrotransformation efficiency under general laboratory conditions. The optimized TG1 transformation system might facilitate large‐scale gene transduction for phage display library construction.

## Graphical abstract

Certain parameters for the preparation of TG1 competent cells include optimum culture time of monoclonal bacteria, amplification growth concentration, and culture volume. The optimized transformation efficiency of TG1 (≥8 × 10^10^ cfu/μg DNA) was observed under suitable electric voltage (2.5 kV), electric intensity (15 kV/cm), and electric time (3.5 ms) of electricity for plasmid transformation. Optimized DNA amount (0.01–100 ng) dissolved in water led to the high efficiency of plasmid transformation but had low efficiency when dissolved in the T4 ligation buffer.

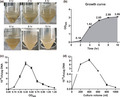

## 1 INTRODUCTION

Phage display library has proven to be a powerful technology for screening of single‐chain antibody fragment (scFv), which provides candidate sequences for the development of fully human monoclonal antibody (mAb) drugs, chimeric antigen receptor (CAR)‐T cell drugs, or antibody‐based therapeutics (Mimmi, Maisano, Quinto, & Iaccino, 2019). The efficient transformation of bacteria is dependent on a universal and convenient technique of a highly efficient electrotransformation system and is crucial to the successful establishment of phage display libraries (Aune & Aachmann, 2010). Various methods of electrotransformation have been developed and optimized with pulse voltage, pulse time, electric intensity, and electroporation buffers to yield different transformation efficiencies (Cui, Smooker, Rouch, & Deighton, 2015; Liu et al., 2014). A previous study demonstrated that the electrotransformation efficiency of *Xanthomonas campestris* prepared under optimal conditions was 10^9^ colony forming units (cfu)/μg for plasmid DNA transformation (Xiuli Wang & Liang, 2016). High electro transformation efficiency of 10^7^ cfu/μg DNA was observed in *Corynebacterium glutamicum* by weakening its cell wall (Li, Zhang, Guo, & Xu, 2016). However, these electro transformation systems are still unsatisfactory for large phage antibody libraries.

The preparation of a high‐quality‐antibody phage display library depends on high‐efficiency gene transfer into competent cells. TG1 is a derivative strain of *Escherichia coli* JM101, which has neither modification nor restriction on transformed exogenous DNA. Currently, TG1 might be the fastest‐growing clone of *E*. *coli* strains, visualized in the LB plate after approximately 7 hr at 37°C. Therefore, TG1 electrocompetent cells are considered an ideal selection for gene introduction in large phage libraries (Clackson, Hoogenboom, Griffiths, & Winter, 1991). The transformation efficiency of TG1electrocompetent cells was influenced by DNA amount, cell growth stage, field strength, and recovery time (Chen, Guo, Xie, & Shen, 2001). It was previously reported that the electrotransformation efficiency of *E. coli* is up to 10^8^–10^9^ cfu/μg DNA in a general laboratory (Tu et al., 2005), in which electrocompetent cells are less effective and unable to meet the requirements of large phage antibody libraries. Furthermore, commercial companies (such as Lucigen) have higher efficient TG1 cells available, at ≥4 × 10^10^ cfu/µg DNA. However, these cells are expensive and the transportation process leads to temperature fluctuations, thereby reducing efficiency. Therefore, the development of a high‐efficient electrotransformation system is urgently needed for phage display antibody libraries.

To establish the high‐efficient electrotransformation system, we optimized conditions for TG1 competent cell preparation and the parameters of electrotransformation. A highly efficient system for pUC19 by electrotransformation of TG1 has been developed by optimizing culture time of monoclonal bacteria (8–10 hr), the concentration of bacteria (OD = 0.45), culture volume (400 ml in 2 L conical flask), recovery time (1.5–2 hr) of electrotransformation, and voltage (2.5 kV), time (3.5 ms), and intensity (15 kV/cm) of electricity. Furthermore, DNA amount and agents also affect the efficiencies tested in water, T4 buffer. Together with the optimized effects of TG1 on the electrotransformation system, transformation efficiency reached ≥8 × 10^10^ cfu/μg of plasmid DNA. Therefore, higher electrotransformation efficiency of the optimized TG1 transformation system could meet the need of phage display library construction.

## 2 MATERIALS AND METHODS

### 2.1 Bacterial strains and Plasmids

The *E. coli* TG1 was from iCARTAB biomedical co. LTD. The plasmids (pUC19 and pCanTab‐5F) used in this study were obtained from Takara Bio Inc and iCARTAB biomedical co. LTD and stored in our laboratory.

### 2.2 Reagents

Bacto‐Tryptone, Bacto‐Yeast, Extract, glucose, and Hepes were purchased from Sigma; Glycerol, Glucose, Sodium chloride (NaCl), Magnesium chloride (MgCl_2_), Magnesium sulfate (MgSO_4_), Sodium hydroxide (NaOH), and other reagents were of analytical reagent grade and purchased from Sinopharm Chemical Reagent (Shanghai, China); T4 DNA ligase buffer was purchased from NEB (Beijing, China).

### 2.3 Media for bacterial growth

#### 2.3.1 Luria‐Bertani (LB) medium

Ten grams per liter Bacto‐Tryptone, 5 g/L Bacto‐Yeast Extract, and 5 g/L NaCl, adjusted to a pH of 7.5 with NaOH were sterilized in an autoclave. The medium was allowed to cool to 55 ºC, after which ampicillin (final concentration 100 μg/mL) was added.

#### 2.3.2 LB medium plates

Fifteen grams per liter of 1.5% Bacto‐agar was added to the LB medium before autoclaving. For the selection of transformed *E*. *coli*, LB plates containing 100 μg/mL of ampicillin were used.

#### 2.3.3 2× yeast extract tryptone (YT) media

Five grams of NaCl, 10 g Bacto Yeast Extract, and 16 g Bacto Tryptone were dissolved in 1 L of ddH_2_O, and sterilized in an autoclave; SOC medium: Two percent Bacto‐Tryptone, 0.5% Yeast Extract, 10 mM NaCl, 2.5 mM MgCl_2_, 10 mM MgSO_4_, 20 mM glucose (pH 7.0).

### 2.4 Plotting TG1 growth curves

The frozen stock of TG1 was streaked with a sterilized inoculation loop onto an LB medium plate and was incubated overnight at 37°C. Thereafter, 5 ml of LB medium in a 50 ml flask was inoculated with a single colony of TG1. The flask was incubated on a shaking platform at a speed of 250 rpm at 37°C for 8–10 hr. The culture concentration of TG1 was measured at 2 hr intervals from the time of inoculation (0 hr) through a 10 hr incubation period. Growth curves were plotted according to the absorbance recorded at 600 nm.

### 2.5 Preparation of electroporation‐competent cells

A frozen stock of TG1 was inoculated onto an LB Medium plate using a sterilized inoculation loop and incubated overnight at 37°C. A single TG1 colony was inoculated into 5 ml of LB medium in a 50 ml flask. The flask was incubated on a shaking platform with a speed of 250 rpm at 37°C for 8–10 hr. A 5 ml overnight culture of TG1 was inoculated into 2 L baffled flasks containing 400 ml of 2 × YT medium for a shaking culture (250 rpm, 37°C). After 1.5–2 hr, 1 ml of each culture was transferred to plastic cuvettes and the optical density measured at OD_600_. At OD_600_ = 0.45, the cultures were transferred to sterile, prechilled (4°C) 250 ml centrifuge bottles. The bottles were placed on ice for 30 min and then centrifuged at 7,000 rpm for 10 min at 4°C in a centrifuge (Thermo Fisher Scientific). The supernatants were discarded and cell pellets resuspended by gentle trituration in 250 ml of ice‐cold 4‐(2‐hydroxyethyl)‐1‐piperazineethanesulfonic acid (HEPES) solution (pH 7.0). After centrifugation, supernatants were discarded and cell pellets pooled with 50 ml ice‐cold sterile 10% (v/v) glycerol in a 50 ml centrifuge tube. The supernatant was discarded and the cell pellet gently resuspended in a total volume of 3 ml 10% glycerol. Cells were either used immediately for transformation or frozen in liquid nitrogen, and subsequently stored at −80°C.

### 2.6 TG1 electrotransformation

The LB plates and SOC medium with Ampicillin (100 μg/mL) or Kanamycin (50 μg/mL) were preheated at 37 ºC for 1 hr. Fifty microliters of TG1 electroporation‐competent cells were thawed on ice; to which 1 μL plasmid DNA was added. Thereafter, cells were mixed gently, and the cell‐DNA mixture was transferred to a chilled sterile electroporation cuvette (0.2 cm gap). Cuvettes were tapped until the mixture settled evenly at the bottom and placed on ice for 5 min. Electroporation on the electroporator (Bio‐Rad) was performed using optimized parameters (voltage setting of 2.5 kV, resistance at 200 Ω and capacitance at 25 µF). The cuvette was placed into the electroporation chamber until it sat flush against the electrical contacts. The sample was pulsed and the cuvette quickly removed and 950 µl of SOC medium was immediately added to resuspend the cells. Subsequently, cells were transferred to a sterile 15 ml tube (BD Biosciences) and incubated at 37°C for 1–2 hr with shaking at 250 rpm in a shaker incubator. A suitable dilution of the mixture was spread onto a preheated plate containing antibiotics and incubated at 37°C overnight to allow the growth of monoclonal colonies, that would be counted later.

### 2.7 Calculation of electrotransformation efficiency

The electrotransformation efficiency was calculated as cfu/μg of plasmid DNA to TG1 cells. The computational formula was as follows:

$$\text{Transformation efficiency}\;\left(\text{cfu}/\mu g\right)=\frac{N\times 10^{3}\times 10^{n}}{v\times C\times V\times 10^{-3}}$$

**N**, The number of average clones; **n**, Dilution ratio; **v**, Point sample volume (μL); **C**, DNA concentration (ng/μL); **V**, Recovery medium volume (mL).

### 2.8 Statistical analysis

Data were represented as the mean standard deviation (mean ± *SD*) and analyzed using GraphPad Prism software (La Jolla, CA). Two‐tailed independent Student's *t* test was performed and *p < .05; **p < .01; ***p < .001 were used to indicate various statistical significance levels.

## 3 RESULTS

### 3.1 Optimization of culture condition enhances the transformation efficiency of TG1 electrocompetent cells

**FIGURE 1:**
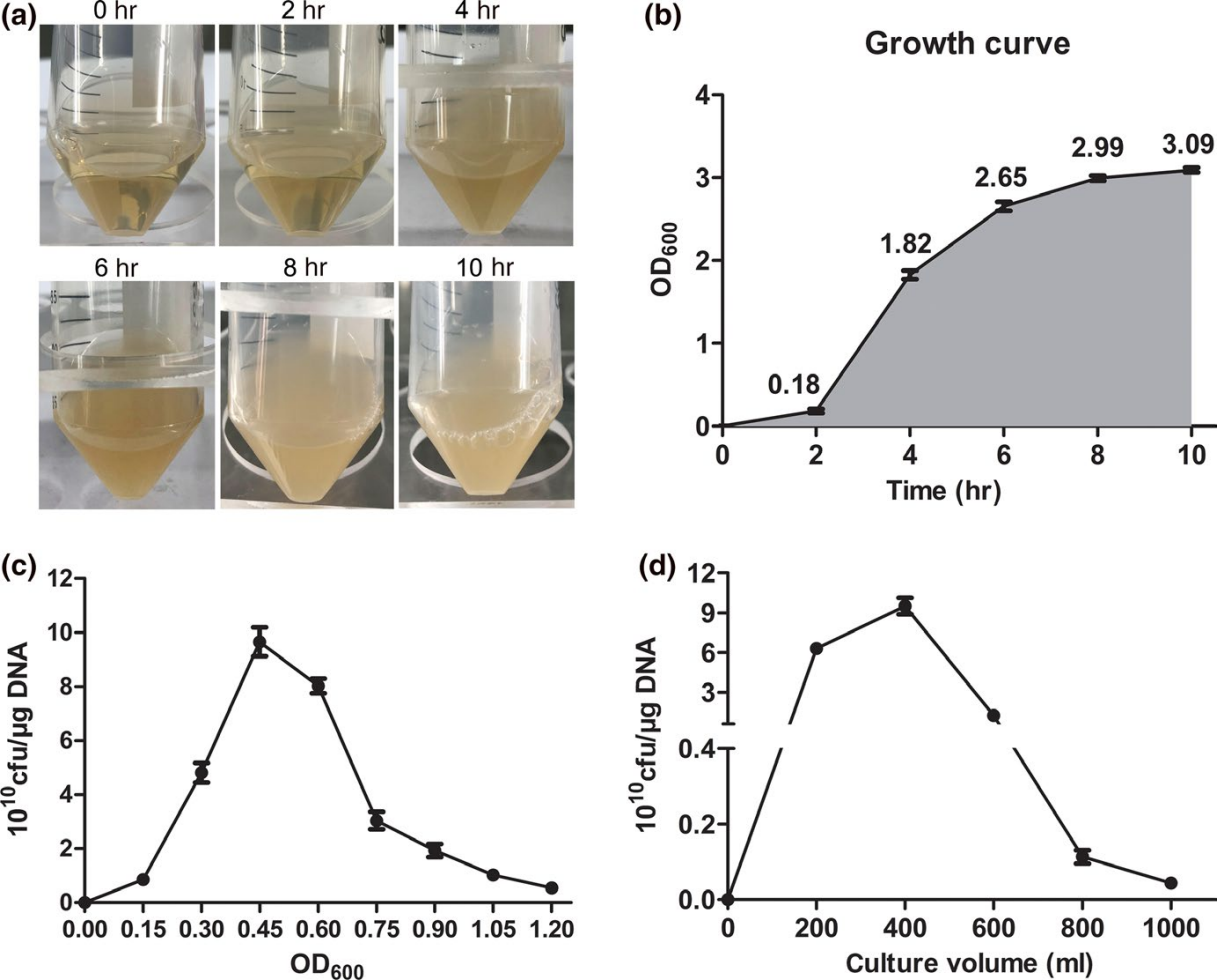
Effects of culture conditions on electrotransformation efficiency in TG1. (a) Images of TG1 culture in LB medium at different times. (b) The bacterial optical density (OD_600_) was determined using a bio‐photometer in (a). (c) The electrotransformation efficiency of cell growth stages. Cells were collected at different stages to generate competent cells, ranging from OD_600_ = 0–1.2. (d) Electrotransformation efficiency in TG1 competent cells from various culture volumes. Other parameters were cells of OD_600_ = 0.45, electrodes of 0.2 cm gap, 3.5 ms of time, and 2 hr recovery time. One nanogram of plasmid pUC19 was used. All experiments were performed in triplicate. Data are represented as the mean ± *SD*

The cell growth period of monoclonal bacteria played an essential role in influencing growth activity for the expanded culture of bacteria. Incubation time is referred to as the cultivation time after a foreign plasmid was introduced into bacteria. Furthermore, the value of OD_600_ reflects the growth status. For general transformation experiments, the OD values required are between 0.3–0.5 and the incubation time is less than 2 hr (Dagert & Ehrlich, 1979; Inoue, Nojima, & Okayama, 1990; Liu et al., 2018). The incubation time for a general transformation experiment should not be too long, as it can easily decrease transformation efficiency. As shown in Figure 1a,b, TG1 values showed that cell growth and density reached a plateau (8–10 hr), and OD_600_ reached about 2.99 at 8 hr. These results indicate that the optimization growth period of monoclonal bacteria is approximately 8–10 hr. Generally, the determination of the early‐log phase of bacteria required for competent cell preparation is important to improve transformation efficiency. When compared with other groups, we observed that the OD_600_ at approximately 0.45 had the highest transformation efficiency (≥8 × 10^10^ cfu/μg DNA) (Figure 1c). Also, TG1 bacterial growth requires ample oxygen and space. We observed that 400 ml of TG1 medium cultured in a 2 L flask obtained the optimum transformation efficiency (Figure 1d). The results showed that optimal incubation times of monoclonal bacteria, bacterial growth concentrations, and culture volumes could result in high transformation efficiency.

### 3.2 Storage time of TG1 electrocompetent cells and recovery treatment time after transformation affect transformation efficiency

**FIGURE 2:**
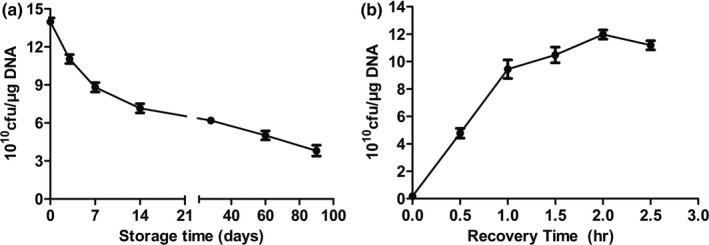
Effects of competent cell storage time and recovery treatment time on transformation efficiency. Replicative plasmid pUC19 was used in these experiments. (a) Effect of storage time on TG1 competent cells. The storage time ranged from 0–90 d. (b) The electrotransformation efficiency in TG1 competent cells from various recovery treatment times. Other parameters were cells of OD_600_ = 0.45, electrodes of 0.2 cm gap, 3.5 ms time, and 2 hr recovery time. One nanogram of plasmid pUC19 was used and the other parameters were the same as above. All experiments were performed in triplicate. Data are represented as the mean ± *SD*

To analyze whether different factors influence the transformation efficiency of TG1 electrocompetent cells, the storage and recovery times after transformation were investigated. With prolonged storage time, transformation efficiency of TG1 competent cells gradually reduced in transformation ability, represented by a reduced number of colonies on the plate (Figure 2a). However, by the 90th day, electrotransformation efficiency was ≥10^10^ cfu/μg DNA. After transformation, recovery treatment is necessary for cells to recuperate from osmotic stress damage, cell division or internal restrictions, thus increasing the number of selected single colonies. As shown in Figure 2b, the recovery treatment time has an enhancing effect on transformation efficiency. Higher electrotransformation efficiency was observed at the optimal recovery time which is approximately 2 hr. When the recovery treatment time was prolonged, transformation efficiency increased. However, this process does not require too long of a time, as this can lead to a false‐positive result.

Consequently, we can conclude that freshly prepared TG1 electrocompetent cells and a 2 hr recovery time after transformation are necessary for optimal transformation efficiency. In addition, recovery treatment is beneficial to stable expression of exogenous genes, and the addition of any antibiotics in recovery medium should be avoided as it can affect the transformation efficiency of competent cells.

### 3.3 Effect of the electrotransformation parameters on transformation efficiency of TG1 competent cells

**FIGURE 3:**
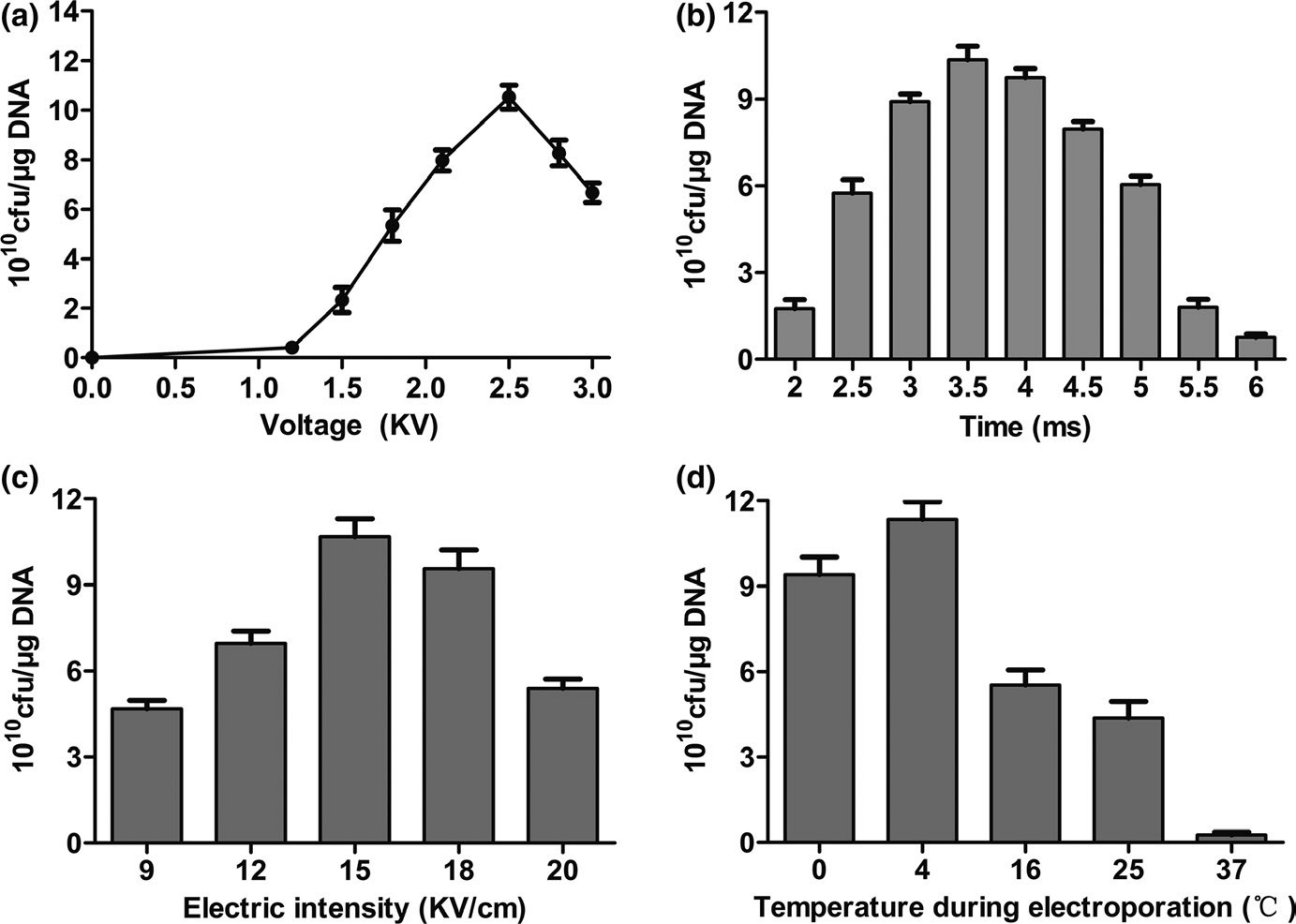
Effects of different parameters on electrotransformation efficiency in TG1 competent cells. Replicative plasmid pUC19 was used in these experiments. (a) Effect of voltage; (b) Effect of time; (c) Effect of intensity; (d) Effect of temperature during electroporation. Other parameters were cells of OD_600_ = 0.45, electrodes of 0.2 cm gap, and 2 hr recovery time. One nanogram of plasmid pUC19 was used, and the other parameters were the same as above. All experiments were performed in triplicate. Data are represented as the mean ± *SD*

Different electrotransformation parameters were used for testing transformation efficiency, showing different transformation efficiencies on competent cells. TG1 competent cells prepared under optimal conditions had the highest transformation efficiency at the appropriate voltage (2.5 kV) (Figure 3a). On the other hand, transformation efficiency of TG1 was completely different at varying times, the highest being at 3.5 ms (Figure 3b).Furthermore, we observed that the electric intensity (15 kV/cm) used resulted in the highest transformation efficiency when compared with other groups (Figure 3c). Notably, a temperature of 4°C during electrotransformation showed higher transformation efficiency than that of other groups (Figure 3d). Therefore, these results indicated that optimal electrotransformation parameters could enhance the transformation efficiency of TG1 competent cells.

### 3.4 Effects of DNA amount or T4 buffer on transformation efficiency of TG1 competent cells

**FIGURE 4:**
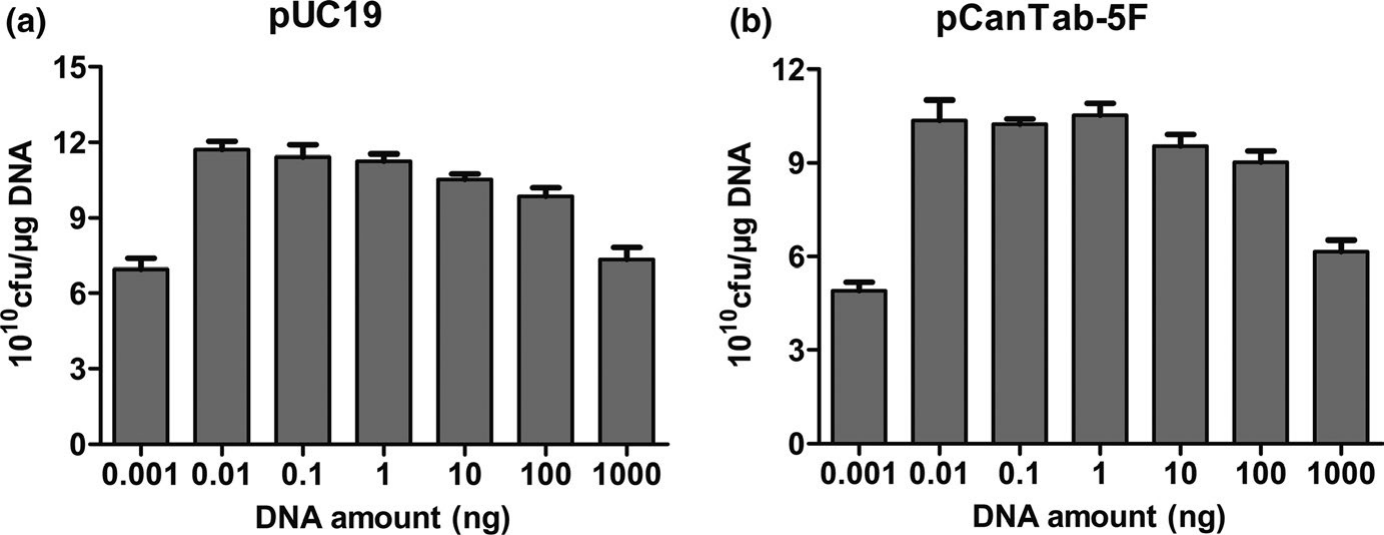
Effect of different amount of DNA during electroporation on transformation efficiency of DNA into TG1. (a): Effect of different pUC19 plasmid concentrations; (b): Effect of different pCanTab‐5F plasmid concentrations; The DNA concentration ranged from 0.001 to 1,000 ng. Other parameters were cells of OD_600_ = 0.45, electrodes of 0.2 cm gap, 3.5 ms time, and 2 hr recovery time. The data shown are representative of three experiments

**FIGURE 5:**
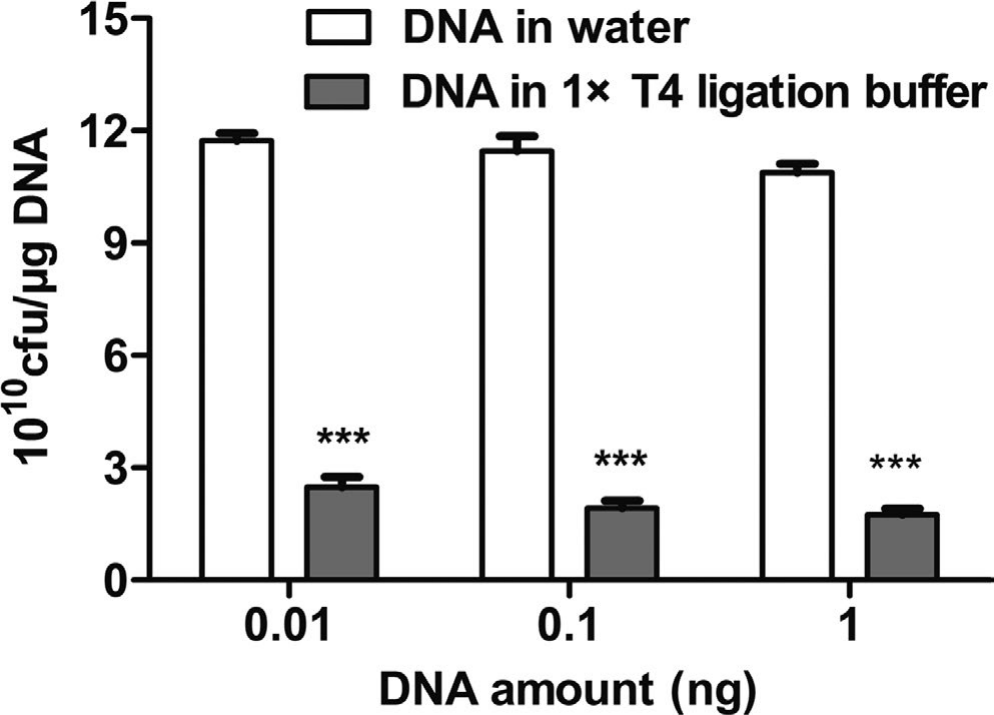
Transformation efficiency of DNA in T4 ligation buffer on TG1. Effect of plasmid pUC19 in T4 ligation buffer; each experiment was performed independently at least three times and the results of one representative experiment are shown. Other parameters were cells of OD_600_ = 0.45, electrodes of 0.2 cm gap, 3.5 ms time, and 2 hr recovery time. Each data represents the mean ± *SD* of three experiments; *p < .05, **p < .01, and ***p < .001 were used to indicate various statistical significance levels

In this experiment, it was hypothesized that an increase in plasmid amount would present a tendency to increase transformation efficiency. Thus, the plasmid amount also plays a key role in the changeable process and the appropriate amount of the plasmid is advantageous to the transformation. We performed the following experiment to find the optimal amount of plasmid DNA for TG1 bacteria. From Figure 4a, we observed that transformation efficiency was at its highest when 0.01–1 ng pUC19 plasmid was added. With an increase in plasmid amount, transformation efficiency had decreased. A higher plasmid amount resulted in toxic effects on cells and had a significant negative effect on transformation efficiency. Of interest, the phage expressing plasmid pCanTab‐5F had a larger molecular weight than plasmid pUC19. Generally, transformation efficiency decreased with the larger size of the plasmid (Figure 4b). Also, saline ions are important factors influencing the transformation efficiency of competent cells. As shown in Figure 5, DNA dissolved in T4 buffer showed a significant decrease in transformation efficiency as compared to DNA dissolved in water. These results indicated that the amount of DNA or T4 buffer affected the transformation efficiency of TG1 competent cells.

## 4 DISCUSSION

The phage antibody display library technology is an efficient screening approach, which provides a molecular diversity tool for creating scFvs and the discovery of new mAb or CAR‐T therapeutics (Srivastava & Riddell, 2015; Zhao, Tohidkia, Siegel, Coukos, & Omidi, 2016). TG1 electrotransformation is widely applied in phage display libraries due to its high efficiency (Cawez et al., 2018). However, due to cost, transportation or electrotransformation efficiency, TG1 competent cells are unable to meet the requirement of large phage antibody display libraries in a general laboratory. This requires optimization or modification in the competent cell preparation and electrotransformation procedures. Our research mainly used the optimal electrotransformation system to obtain a better understanding of a series of factors affecting transformation efficiency and analyzed each of these factors via the electrotransformation experiments.

The culture time of monoclonal bacteria, amplification growth concentration and culture volume play an important role in TG1 viability, which determines the success of the preparation of competent cells. In this study, the optimal electrotransformation system investigated these factors affecting transformation efficiency. Our results showed that the culture time between 8 and 10 hr for monoclonal bacteria is the optimal plateau period for bacterial growth. The amplification growth concentration of approximately 0.45 at OD_600_ can obtain optimal cell viability. A culture volume of 400 ml in 2 L conical flask provided ample space and oxygen for bacterial growth. Therefore, the culture time and the growth concentration of TG1 were the important factors affecting transformation efficiency. Furthermore, freshly prepared competent cells showed the highest transformation efficiency compared with other TG1 at different storage times. This is due to a decrease in bacterial activity as the storage time increases, leading to a decrease in transfection efficiency. Overall, our research provides further insight into the enhancement of transformation efficiency of TG1 (8 × 10^10^ cfu/μg DNA) by optimizing culture time, amplification growth concentration and culture volume.

This study also showed that the recovery time of electrotransformation, along with voltage, time, and intensity of electricity, plays a key role in the transformation experiment. Our results demonstrated that the advisable recovery treatment time for electrotransformation is 1.5–2 hr on a shaking platform with a speed of 250 rpm at 37°C. The transformation efficiency increased 10‐fold after 2 hr than in 1 hr. However, it is prone to form false‐positive clones that can be avoided by correctly increasing the incubation time. Therefore, a minimum 1 hr and maximum 2 hr recovery time is necessary for high transformation efficiency of TG1. As acknowledged, higher voltage, time, and intensity of electricity resulted in the death of large numbers of TG1 competent cells, thereby decreasing transformation efficiency (Janez, Meglic, Flisar, Miklavcic, & Peterka, 2019). In this study, the optimal electrotransformation system including voltage (2.5 kV), time (3.5 ms), and intensity (15 kV/cm) resulted in the highest transformation efficiency.

Also, the amount of DNA or dilution buffer had a substantial impact on transformation efficiency. When the optimal amount of DNA (0.01–100 ng) was diluted in water on a selective plate immediately after electroporation, the transformation efficiency was ≥8 × 10^10^ cfu/μg DNA, but the plasmid diluted in T4 ligation buffer had a transformation efficiency of approximately ≤3 × 10^10^ cfu/μg DNA. This is because the ions in the buffer reduce the voltage and current of the electrical transformation, resulting in the decreased efficiency of cell perforation, resulting in the low efficiency of electrotransformation.

In conclusion, high‐efficient electrotransformation system including TG1 electrocompetent cells was feasible to generate efficient electrocompetent cells; combined with optimized electroporation parameters to increase transformation efficiency for the plasmid (≥8 × 10^10^ cfu/μg DNA). Thus, our optimized TG1 transformation system might be applied for high electrotransformation efficiency under normal laboratory conditions and can facilitate the construction of a phage display library.

## CONFLICT OF INTEREST

None declared.

## AUTHORS' CONTRIBUTIONS

Dafei Chai: Conceptualization (equal); Data curation (equal); Formal analysis (equal); Investigation (equal); Methodology (equal); Writing‐original draft (equal). Gang Wang: Data curation (equal); Formal analysis (equal); Investigation (equal); Software (equal); Validation (equal). Lin Fang: Investigation (equal); Methodology (equal). Huizhong Li: Investigation (equal); Methodology (equal). Shanshan Liu: Investigation (equal); Methodology (equal). Haiying Zhu: Investigation (equal); Methodology (equal). Junnian Zheng: Conceptualization (equal); Project administration (equal); Resources (equal); Supervision (equal); Writing‐original draft (equal).

## ETHICS STATEMENT

None required.

## Data Availability

All data generated or analyzed during this study are included in this published article.
